# *Trypanosoma brucei brucei* Induces Polymorphonuclear Neutrophil Activation and Neutrophil Extracellular Traps Release

**DOI:** 10.3389/fimmu.2020.559561

**Published:** 2020-10-22

**Authors:** Daniela Grob, Iván Conejeros, Zahady D. Velásquez, Christian Preußer, Ulrich Gärtner, Pablo Alarcón, Rafael A. Burgos, Carlos Hermosilla, Anja Taubert

**Affiliations:** ^1^Institute of Parasitology, Biomedical Research Center Seltersberg (BFS), Justus Liebig University Giessen, Giessen, Germany; ^2^Institute of Biochemistry, Department of Biology and Chemistry, Justus Liebig University Giessen, Giessen, Germany; ^3^Institute of Anatomy and Cell Biology, Justus Liebig University Giessen, Giessen, Germany; ^4^Laboratory of Inflammation Pharmacology, Institute of Pharmacology and Morphophysiology, Universidad Austral de Chile, Valdivia, Chile

**Keywords:** *Trypanosoma brucei brucei*, NETs formation, PMN, *agg*NETs, purinergic receptors

## Abstract

*Trypanosoma brucei brucei* trypomastigotes are classical blood parasites of cattle, these stages might become potential targets for circulating polymorphonuclear neutrophils (PMN). We here investigated NETs extrusion and related oxygen consumption in bovine PMN exposed to motile *T. b. brucei* trypomastigotes *in vitro*. Parasite exposure induced PMN activation as detected by enhanced oxygen consumption rates (OCR), extracellular acidification rates (ECAR), and production of total and extracellular reactive oxygen species (ROS). Scanning electron microscopy (SEM) showed that co-cultivation of bovine PMN with motile trypomastigotes resulted in NETs formation within 120 min of exposure. *T. b. brucei*-induced NETs were confirmed by confocal microscopy demonstrating co-localization of extruded DNA with neutrophil elastase (NE) and nuclear histones. Immunofluorescence analyses demonstrated that trypomastigotes induced different phenotypes of NETs in bovine PMN, such as aggregated NETs (*agg*NETs), spread NETs (*spr*NETs), and diffuse NETs (*diff*NETs) with *agg*NETs being the most abundant ones. Furthermore, live cell 3D-holotomographic microscopy unveiled detailed morphological changes during the NETotic process. Quantification of *T. b. brucei*-induced NETs formation was estimated by DNA and nuclear area analysis (DANA) and confirmed enhanced NETs formation in response to trypomastigote stages. Formation of NETs does not result in a decrease of *T. b. brucei* viability, but a decrease of 26% in the number of motile parasites. Referring the involved signaling pathways, trypomastigote-induced NETs formation seems to be purinergic-dependent, since inhibition via NF449 treatment resulted in a significant reduction of *T. b. brucei*-triggered DNA extrusion. Overall, future studies will have to analyze whether the formation of *agg*NETs indeed plays a role in the outcome of clinical disease and bovine African trypanosomiasis-related immunopathological disorders, such as increased intravascular coagulopathy and vascular permeability, often reported to occur in this disease.

## Introduction

Animal African trypanosomiasis (AAT), also known as Nagana, has been recognized as a devastating and still neglected cattle disease in sub-Saharan Africa for centuries (1). The causal agent of AAT is the haemoflagellate parasite *Trypanosoma brucei brucei*, being transmitted by blood-meal bites of female tsetse flies (*Glossina* spp.). It is still considered as a major cause of mortality and morbidity in domestic cattle, sheep, goats, and horses. Pathogenesis of AAT is complex and starts with primary, localized inflammatory lesions at the site of *T. b. brucei* inoculation after successful tsetse bites, followed by intensive local asexual parasite multiplication and dissemination of the trypomastigote stage via lymphatic and blood vessels to regional lymph nodes, internal organs, central nervous system, cerebellum, and spinal cord (2–4). Consequently, clinical manifestations of AAT include generalized lymphadenopathy, splenomegaly, increased vascular permeability, edema, haemostasis, intravascular coagulopathies, anemia, tissue hypoxia, formation of immune complexes, glomerulonephritis, severe immunosuppression, and sudden death. *T. b. brucei*-infected cattle with or without clinical symptoms are considered as the main parasite reservoirs for AAT in Africa (5).

In the sub-Saharan African region, AAT in domestic livestock causes reduction of meat and milk production, restraining the labor function of *T. b. brucei*-infected animals, thereby causing high economic losses. Accordingly, economic benefits of tsetse fly elimination programs have been estimated for up to US$ 4.5 billion per year by avoiding the death of 3 million cattle per year, as well as sheep, horses, and goats. In terms of zoonotic potential, the closely related species *T. brucei gambiense* and *T. brucei rhodesiense* are the causative agents of human African trypanosomiasis (HAT) or sleeping sickness, which is lethal if untreated and classified as well as a neglected tropical disease by the World Health Organization (WHO) (6).

*In vivo*, direct contact of *T. b. brucei* stages with leukocytes of the host innate immune system occurs during the parasite-endogenous replication phase, for example, (i) after initial tsetse bite-mediated inoculation of procyclic trypomastigotes into the skin, (ii) when metacyclic trypomastigotes enter the lymphatic/blood vessels, and (iii) when metacyclic trypomastigotes replicate in diverse organs. Polymorphonuclear neutrophils (PMN) are the most abundant leukocyte population in lymph and bloodstream and rapidly recruited from circulation to sites of infection (7–9). In this context, local pro-inflammatory responses in skin lesions in AAT, resulting in focal edema, were associated with PMN recruitment and granuloma formation surrounding *T. b. brucei* replication sites (10). PMN reacts against protozoan and metazoan parasites by different effector mechanisms which include the release of immunomodulatory molecules [e.g., cytokines, chemokines (CXCL1, CXCL8, CXCL10) (11, 12)], phagocytosis, production of reactive oxygen species (ROS), and release of neutrophil extracellular traps (NETs) (11, 13, 14). So far, different parasite species were identified to induce either nicotinamide adenine dinucleotide phosphate (NADPH) oxidase (NOX)-dependent or NOX-independent NETs formation (15, 16). During parasite-triggered NETs release, nuclear chromatin decondensation is facilitated by protein arginine deiminase 4 (PAD4)-mediated citrullination of histones (16–18). NETs-related enzymes, such as neutrophil elastase (NE) and myeloperoxidase (MPO), translocate to the nucleus and fuse with chromatin (19). Finally, PMN membrane disintegration is mediated either by enhanced ROS production (20) or by actions of lytic proteins, such as gasdermin-D, mediating membrane pore formation and subsequent NETs extrusion into the extracellular matrix (21). NETs release is a regulated molecular process which depends on energy metabolism (22, 23), activation of NOX and generation of ROS and Ca^++^-influx as second messengers among others (9). Overall, the NETotic process can occur in a NOX-dependent and a NOX-independent mode, being classified as suicidal NETosis, vital NETosis or vesicular NETosis, respectively (24). Suicidal NETosis includes the stimulation of PMN, translocation of NE and MPO into nucleus resulting in degradation of nuclear histones and PAD4-mediated chromatin decondensation after the disintegration of the nuclear membrane and final PMN death (7, 19, 20, 24). In contrast, vital NETosis is described as a process in which PMN not necessarily will die. Here, PMN release NETs from mitochondrial origin without losing cell vitality, being nowadays addressed as non-lytic NETs (16, 24–26).

Zoonotic relevant euglenozoan parasites, such as *Leishmania* spp. and *Trypanosoma cruzi*, were recently described to trigger NETs release in different hosts, such as humans, mice, opossums and dogs, evidencing NETs formation as an ancient and evolutionary well-conserved innate effector mechanism among mammalian species (4, 11, 13, 14, 16, 27–30). So far, data on the role of NETs against highly motile *T. b. brucei* trypomastigotes are entirely lacking.

Data on metabolic requirements of PMN during parasite-triggered NETs formation are limited (31). Nevertheless, it is known that extracellular adenosine 5′-triphospate (ATP) availability and activation of P2 purinergic receptors play fundamental roles in PMN activation (31). Thus, P2-mediated purinergic signaling pathways are involved in the regulation of essential functions of PMN, such as chemotaxis, phagocytosis, oxidative burst, degranulation (31). Consistently, P2-mediated purinergic pathways seem crucial in *Neospora caninum-* and *Besnoitia besnoiti*-mediated NETosis (15, 23). Considering these data, we here aimed to evaluate the role of purinergic signaling in *T. b. brucei* triggered NETs formation.

In the current work, PMN activation was estimated by analysis of oxygen consumption rates (OCR), extracellular acidification rates (ECAR), and ROS production. Also, we observed by using scanning electron microscopy (SEM), confocal- and live cell three-dimensional (3D) holotomographic microscopy that exposure of bovine PMN with trypomastigotes resulted in the formation of different phenotypes of NETs *in vitro*. Quantification of *T. b. brucei*-triggered NETs release was performed by DNA and nuclear area expansion (NAE) [“DNA area and NETosis analysis” (DANA)] analysis; *T. b. brucei*-triggered NETs formation also revealed as purinergic-dependent, since PMN treatment with the inhibitor NF449 decreases the release of extracellular DNA.

## Materials and Methods

### Ethics Statements

This study was conducted following the Justus Liebig University Giessen (JLU) Animal Care Committee Guidelines. Protocols were approved by Ethics Commission for Experimental Animal Studies of Federal State of Hesse (Regierungspräsidium Giessen; A9/2012; JLU-No.521_AZ) and in accordance to European Animal Welfare Legislation: ART13TFEU and current applicable German Animal Protection Laws.

### Parasites

*Trypanosoma brucei brucei* trypomastigotes of the strain 427 (32) were grown on plastic T-25 cm^2^ tissue culture flasks (Greiner) in SDM-79 cell culture medium (General Electric Health) supplemented with 10% fetal calf serum (FCS; Greiner) for parasite proliferation. The medium was changed every 4 days, as described by Cross and Manning (33).

### Isolation of Bovine PMN

Healthy adult dairy cows (*n* = 9) served as blood donors. Blood was obtained by puncture of the jugular vein and 30 ml was collected in 12 ml heparinized sterile plastic tubes (Kabe Labortechnik). Then, 20 ml of heparinized blood was diluted in 20 ml sterile phosphate-buffered saline (PBS) with 0.02% ethylenediaminetetraacetic acid (EDTA) (Sigma-Aldrich), layered on top of 12 ml Biocoll® separating solution (density = 1.077 g/l; Biochrom AG) and centrifuged (800 × g, 45 min). After the removal of plasma and peripheral blood mononuclear cells (PBMC), cells were suspended in sterile 25 ml bi-distilled water and gently mixed during 40 s to lyse erythrocytes. Osmolarity was rapidly restored by adding 4 ml of 10x Hanks balanced salt solution (HBSS; Biochrom AG). For complete erythrocyte lysis, this step was repeated twice and bovine PMN were later suspended in sterile RPMI 1640 cell culture medium (Sigma-Aldrich). PMN were counted in a Neubauer haemocytometer. Finally, freshly isolated bovine PMN were allowed to rest at 37°C and 5% carbon dioxide (CO_2_) atmosphere for 30 min before experimental use.

### Quantification of Oxygen Consumption Rates and Extracellular Acidification Rates in *T. b. brucei*-Exposed Polymorphonuclear Neutrophils

Activation of bovine PMN was monitored using Seahorse XF® analyzer (Agilent). Briefly, 1 × 10^6^ PMN from three donors were pelleted at 500 × g for 10 min at room temperature (RT). After removal of the supernatant, cells were re-suspended in 0.5 ml of XF® assay medium (Agilent) supplemented with 2 mM of _L_-glutamine, 1 mM pyruvate, and 10 mM glucose. 1 × 10^5^ cells, corresponding to 50 μl of the cell solution, were gently placed in each well of an eight-well XF® analyzer plate (Agilent) pre-coated for 30 min with 0.001% poly-_L_-lysine (Sigma-Aldrich). Then, 50 μl of XF® assay medium (Agilent) were added to blank wells (= no cell-controls). Finally, 130 μl of XF assay medium (Agilent) was added to all wells (180 μl total volume) and cells were incubated at 37°C without CO_2_ supplementation for 45 min before Seahorse XF® analyzer measurement. When rotenone/antimycin A was used to inhibit mitochondrial complexes I and III, a 5 μM solution was added to the respective port and injected previously to the addition of *T. b. brucei* trypomastigotes (final concentration 0.5 μM). On the other hand, *T. b. brucei* trypomastigotes were suspended in XF assay medium (Agilent, 300,000 parasites/20 μl) and placed in one of the four injection ports of the instrument. For PMN controls, only 20 μl of XF® assay medium (Agilent) was dispensed. The metabolic assay included basal measurement of three readings followed by injection of vital trypomastigotes or medium and 30 readings over time. The total assay duration was 240 min. Background subtraction, determination of OCR, ECAR, and the area under the curve (AUC) of obtained registries were performed by using Wave® software (Desktop Version, Agilent).

### Estimation of Extracellular and Total Reactive Oxygen Species Production

Estimation of total ROS production was achieved as described for bovine PMN by (34). Briefly, 2 × 10^5^ PMN were stimulated with 3 × 10^5^ *T. b. brucei* trypomastigotes in the presence of 500 μm of luminol for total ROS production and 100 μM of isoluminol in the presence of 4 U/ml horseradish peroxidase (HRP) to evaluate extracellular superoxide production. After stimulation, luminescence was monitored every 30 min for 120 min using a luminometer (Promega Glomax). Data are presented as relative chemiluminescence units (RLU).

### Scanning Electron Microscopy (SEM) Analysis

Bovine PMN (*n* = 3) were co-cultured with vital *T. b. brucei* trypomastigotes (ratio 1:3) for 120 min on coverslips (10 mm of diameter; Thermo Fisher Scientific) pre-coated with 0.01% poly-_L_-lysine (Sigma-Aldrich) at 37°C and 5% CO_2_. After incubation, cells were fixed in 2.5% glutaraldehyde (Merck), post-fixed in 1% osmium tetroxide (Merck), washed in distilled water, dehydrated, critical point dried by CO_2_-treatment and sputtered with gold particles. Finally, all samples were visualized via a Philips XL30® scanning electron microscope at the Institute of Anatomy and Cell Biology, JLU Giessen, Germany.

### *T. b. brucei*-Triggered NETs Visualized by Immunofluorescence Analysis

Bovine PMN (*n* = 3) were co-cultured with *T. b. brucei* trypomastigotes (ratio 1:3) for 120 min (37°C and 5% CO_2_ atmosphere) on coverslips (15 mm diameter, Thermo Fischer Scientific) pre-treated with 0.01% poly-_L_-lysine (Sigma-Aldrich). After corresponding incubation time, the cells were fixed in 4% paraformaldehyde (Merck) and stored at 4°C until further use. To visualize NETs structures, Sytox Orange® (1:1,000, Life Technologies) was used to stain extracellular DNA, anti-histones (H1, H2A/H2B, H3, and H4, 1:500, Merck #MAB3422) and anti-neutrophil elastase (NE) (1:500, Abcam #ab68672) antibodies were used to detect NETs-specific components/proteins. Therefore, fixed samples were washed three times with sterile PBS and blocked (60 min, RT) in 2% bovine serum albumin (BSA; Sigma-Aldrich) containing 0.3% Triton X-100 (Thermo Fischer Scientific) and incubated in primary antibody solutions for 120 min at RT. After incubation, three washing steps were performed with sterile PBS and incubated in secondary antibody solutions (Alexa 488 goat anti-mouse IgG #A110011, Alexa 405 goat anti-rabbit IgG #A31556, 1:500, Invitrogen) for 120 min at RT in complete darkness. Finally, the samples were washed three times with sterile PBS and mounted upside-down with Fluoromount G® (Thermo Fischer Scientific). Visualization of NETs formation was achieved using an inverted IX81® epifluorescence microscope equipped with an XM 10® digital camera (both Olympus) or by applying confocal microscopy (Zeiss LSM 710®).

### Analysis of Neutrophil Extracellular Traps Phenotypes

For quantification of different NETs phenotypes [i.e., spread NETs (*spr*NETs), diffuse NETs (*diff*NETs), and aggregated NETs (*agg*NETs)] we followed the description by (35, 36). Briefly, bovine PMN (*n* = 3) were seeded (2 × 10^5^/sample, in duplicates) on 0.01% poly-_L_-lysine (Sigma-Aldrich) pre-coated coverslips (Thermo Fischer Scientific) and exposed to *T. b. brucei* trypomastigotes (6 × 10^5^, 1:3 PMN: parasite ratio) for 120 min (37°C, 5% CO_2_). Afterwards, samples were fixed in 2% paraformaldehyde (Merck) and stored at 4°C until further analysis. The phenotypes *spr*NETs, *diff*NETs, and *agg*NETs were visualized by staining extracellular DNA with Sytox Orange® (5 μM, Life Technologies), anti-NE (1:500, Abcam #ab68672), and anti-histones (H1, H2A/H2B, H3, and H4; 1:500, Merck #MAB3422) antibodies as previously described (37, 38). For visual quantification, five random power vision pictures were taken from each experimental condition using an inverted IX81® fluorescence microscope equipped with an XM 10® digital camera (both Olympus) and analyzed microscopically based on typical morphological characteristics according to Muñoz-Caro et al. (36).

### *T. b. brucei* Motility Assays

Motility evaluation of alive *T. b. brucei* trypomastigotes co-cultured with bovine PMN (*n* = 3; ratio 1:3) for 2 h at 37°C and 5% CO_2_ controlled-atmosphere on a 12-well transparent bottom microplate (Falcon). After the incubation time, motility was scored by direct observation using an inverted IX81® phase-contrast microscope (Olympus®). After counting, the results are shown as a percentage of motile trypomastigotes.

### *T. b. brucei* Viability Measurement by Trypan Blue Staining

Viability evaluation of alive *T. b. brucei* trypomastigotes co-cultured with bovine PMN (*n* = 3; ratio 1:3) for 2 h at 37°C and 5% CO_2_ controlled-atmosphere was performed on a 12-well transparent bottom microplate (Falcon). After incubation, Trypan blue staining (Sigma Aldrich) was added to the medium in a 1:10 dilution. After 2 min, viability was evaluated by visual observation using an inverted IX81® microscope (Olympus). The results are expressed as a percentage of alive trypomastigotes.

### Nuclear Area Expansion-Based Quantification of Neutrophil Extracellular Traps-Formation Using DNA Area and NETosis Analysis Software

For NAE-based quantification of *T. b. brucei*-induced NETs formation, DANA I/II software was used following developers recommendations (39). In brief, bovine PMN (*n* = 3) were left in plain medium (RPMI 1640, Sigma-Aldrich) for 30 min and then exposed to *T. b. brucei* trypomastigotes for 120 min at a 1:3 ratio. PMN were then fixed in 2% paraformaldehyde (Merck) and stained with 5 μM Sytox Orange® (Life Technologies) for 30 min at RT. Five images were randomly taken for each condition using an inverted microscope (Olympus IX 81®) and NAE was analyzed using DANA I- and II-software. Cells presenting a decondensed nucleus and exceeding the threshold of 90 μm^2^ of the nuclear area were considered as PMN undergoing NETs formation.

### Live Cell Imaging of *T. b. brucei*-Induced Neutrophil Extracellular Traps Release Using 3D-Holotomographic Microscopy

In total, 1 × 10^6^ isolated bovine PMN were pelleted (300 × g, 10 min, RT). The supernatant was carefully discarded and cells were suspended in 2 ml imaging medium containing 0.1% BSA (Sigma-Aldrich), 2 μM 1,5-bis{[2-(di-methylamino) ethyl]amino}-4,8-dihydroxyanthracene-9,10-dione (DRAQ5) (Thermo Scientific), and 0.5 μM Sytox Green® (Life Technologies). One ml of this cell solution was seeded in an Ibidi® plastic cell plate (35 mm^2^ diameter with low profile) and placed in a top stage incubation chamber (Ibidi) at 5% CO_2_ and 37°C. Resting time of 30 min was used to let PMN settle down in the plastic cell plate. Then, 1.5 × 10^6^ motile *T. b. brucei* trypomastigotes were added to the center of the plastic cell plate. Image acquisition was set for refractive index (RI; 3D tomography), for 4′,6-diamidino-2-phenylindole (DAPI) channel (blue) for DRAQ5 and fluorescein channel (green) for Sytox Green® (Life Technologies) detection, applying time-lapse settings (image acquisition every minute over 180 min) using a Nanolive Fluo-3D Cell Explorer® (Nanolive). At the end of the experiment, each channel was exported separately using Steve® software v.2.6 (Nanolive) and managed with Image J® software (Fiji version 1.7, NIH). In addition, digital staining was performed based on the values of refractive index (RI) of the obtained images.

### Pharmacological Inhibition of Purinergic Receptors and Mitochondrial Activity

Bovine PMN (*n* = 3) were suspended in sterile HBSS buffer (Sigma-Aldrich) at a final concentration of 1 × 10^6^ cells/ml. Sytox Green® (5 μM; Life Technologies) was added and cells were seeded (1 × 10^5^ cells in 50 μl/well) in a 96-well plate transparent bottom microplate (Greiner). The plate was warmed for 30 min at 37°C and thereafter inhibitors were added at a final concentration of 100 μM for NF449 (inhibitor of the purinergic receptors, Tocris, #7038), 1 mM for N-(Methoxysuccinyl)-Ala-Ala-Pro-Val-chloromethyl ketone (CMK; neutrophil elastase inhibitor, Sigma-Aldrich, #M0398), rotenone/antimycin A (inhibitor of complexes I and III, 5 μM, Agilent), and phenylhydrazone (FCCP, disruptor of mitochondrial membrane potential, 0,5 μM, Agilent). Meanwhile, the parasites were pelleted and suspended in sterile HBSS at a concentration of 3 × 10^5^ specimen/50 μl. The kinetic of NETs formation was followed by spectrofluorometric analysis at an excitation wavelength of 504 nm and an emission wavelength of 523 nm by using an automated multiplate monochrome reader (Varioskan Flash® Thermo Scientific) and registered every 2 min for a total of 120 min. Furthermore, for NF449 inhibition studies, bovine PMN, and trypomastigotes were seeded on 0.01% poly-_L_-lysine pre-coated coverslips (Greiner) as previously described. After 120 min of exposure, cells were fixed in 2% paraformaldehyde (Roth) and stained with Sytox Orange® (Life Technologies) for 30 min at RT. Five power vision field images were randomly taken for each condition using an inverted epifluorescence IX81® microscope (Olympus) equipped with a XM 10® digital camera (Olympus) for further analysis.

### Assessment of the Influence of Motility in *T. b. brucei*-Induced DNA Release

Bovine PMN (*n* = 3) were suspended in sterile HBSS buffer (Sigma-Aldrich) at a final concentration of 1 × 10^6^ cells/ml. Sytox Green® (5 μM; Life Technologies) was added and cells were seeded (1 × 10^5^ cells in 50 μl/well) in a 96-well plate transparent bottom microplate (Greiner). The plate was warmed for 30 min at 37°C. Meanwhile, the parasites were heat-inactivated by treatment of 1 h at 50°C, using a Thermomixer 5436 (Eppendorf). After this, alive and heat-inactivated trypomastigotes were pelleted and suspended in sterile HBSS at a concentration of 3 × 10^5^ parasites/50 μl. The kinetic of NETs formation was followed as described previously.

### Statistical Analysis

For all experiments in the current study, except for NAE estimation and inhibitor experiments, statistical significance was defined by a *p*-value of <0.05 by applying non-parametric analyses: Mann-Whitney test when two experimental conditions were compared and Kruskal-Wallis test followed by Dunn's *post-hoc* test for multiple comparisons. Shapiro-Wilk normality test was performed on the data of inhibitors. Differences were estimated by ANOVA. All graphs (mean ± SD), AUC calculations and statistical analyses were performed using Graph Pad® Prism software (v.7.03).

## Results

### *T. b. brucei* Trypomastigotes Induce Activation of Bovine Polymorphonuclear Neutrophils

**Figure 1:**
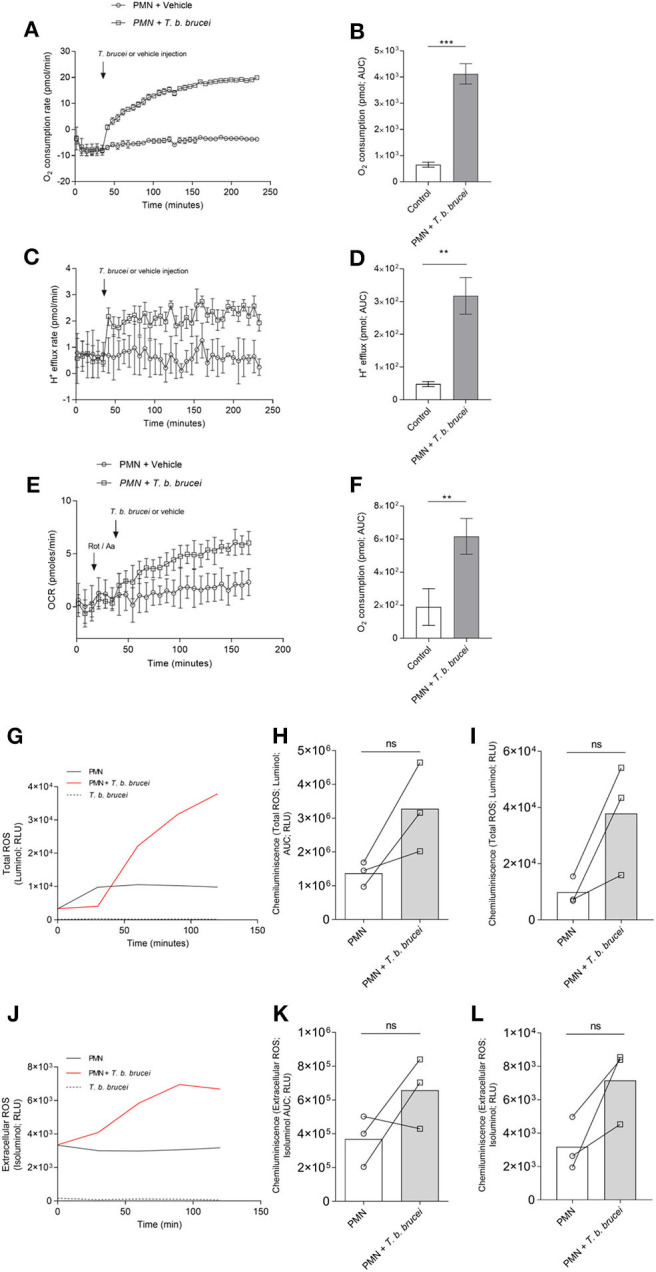
*T. b. brucei*-induced metabolic changes in exposed bovine PMN. Activation of PMN was monitored by an extracellular flux analyzer (Seahorse) for oxygen consumption rate (OCR) proton efflux rate (PER). PMN were incubated in XF RPMI media for 45 min without CO_2_ and then alive *T. b. brucei* trypomastigotes or vehicle was injected at the time point indicated by arrows. The increase in OCR **(A)** and PER **(C)** was monitored for 232 min. The area under the curve (AUC) was calculated for all registries and plotted as mean ± SD (**B,D**; *n* = 3) showing the increase in both parameters for activated PMN. In a slightly different set-up, rotenone/antimycin A was injected before the addition of *T. b. brucei* to inhibit mitochondrial activity (**E,F**; *n* = 5) **p > 0.01, ***p > 0.001 compared with injection of vehicle. Also, total ROS production **(G)** and extracellular superoxide **(J)** was evaluated by the use of luminol and isoluminol, respectively. Both reactive oxygen species increased during the experiment, and the AUC was calculated and represented as mean ± SD. In this case, statistical analysis shows no differences when compared with the control condition (**H,K**; *n* = 3). A similar result is obtained when the luminescence values at 120 min (final reading) are analyzed **(I,L)**.

To evaluate the activation of PMN exposed to *T. b. brucei* trypomastigotes, we performed a series of experiments using Seahorse instrumentation (Agilent). As illustrated in Figure 1, after obtaining basal OCR and proton efflux rates (PER) of plain PMN, live parasites induced a fast and sustained increase in OCR (Figure 1A) and PER (Figure 1C). As shown in Figures 1B,D, analysis of the area under the curve (AUC) revealed a significant increase (*p* < 0.05) in OCR upon parasite exposure. Also, significantly enhanced PER findings (*p* < 0.01) were detected in parasite-exposed PMN when compared to un-stimulated PMN controls. Observed OCR increase was not prevented by rotenone treatment (Figures 1E,F); indicating a contribution of both, NOX and mitochondrial activity, in the increase of OCR in the activation of PMN induced by *T. b. brucei* trypomastigotes. To evaluate if the OCR was linked to ROS production, total ROS, and extracellular ROS production were evaluated in PMN exposed to *T. b. brucei*. Current data shows that *T. b. brucei* induces total and extracellular ROS (Figures 1G,J); however, this increase does not achieve statistical significance when neither AUC (Figures 1H,K) nor the final luminescence value at 120 min (Figures 1I,L) of the obtained registries are analyzed most probably due to inter-individual variation.

### *T. b. brucei* Trypomastigotes Induce Neutrophil Extracellular Traps Formation in Bovine Polymorphonuclear Neutrophils

**Figure 2:**
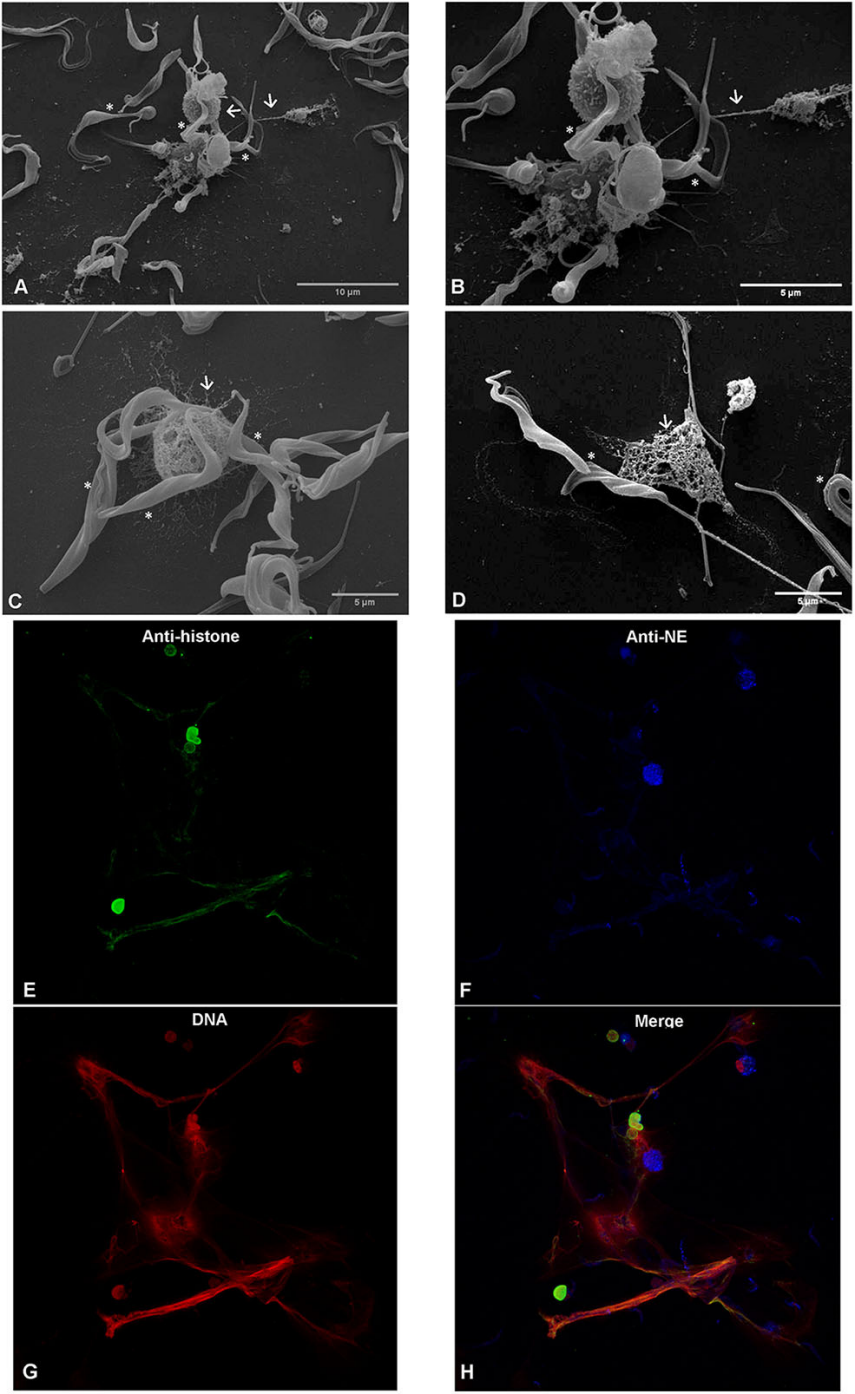
*T. b. brucei*-induced neutrophil extracellular traps (NETs) formation, analyzed via scanning electron microscopy (SEM) and confocal microscopy. Co-culture of bovine polymorphonuclear neutrophils with *T. b. brucei* were PFA-fixed and analyzed by SEM analysis. Co-culture, reveals NETs-like delicate PMN-derived filaroid structures indicated by arrows on **(A–C)**. The presence of the parasite is marked with *. **(D)** Depicts the interactions of NETs derived from one PMN with the *T. b. brucei* flagellum. Confocal microscopy demonstrates co-localization of DNA (**G**; red), histones (H1, H2A/H2B, H3, H4) (**E**; green), and neutrophil elastase (NE) (**F**, blue) in *T. b. brucei* induced NETs structures. **(H)** Corresponds to the merge of the three channels.

SEM analysis unveiled that exposure of bovine PMN to *T. b. brucei* trypomastigotes (asterisks Figures 2A–D) for 120 min induced the formation of both, thick and fine chromatin strands of fibers released from dead PMN (arrows Figures 2A–D). This observation was confirmed as NETs structures since using confocal microscopy, the co-localization of extracellular DNA with histones and NE allowed to confirm the presence of these typical NETs proteins associated with the chromatin released from PMN (Figures 2E–H).

### *T. b. brucei* Trypomastigotes Trigger Different Phenotypes of Neutrophil Extracellular Traps

**Figure 3:**
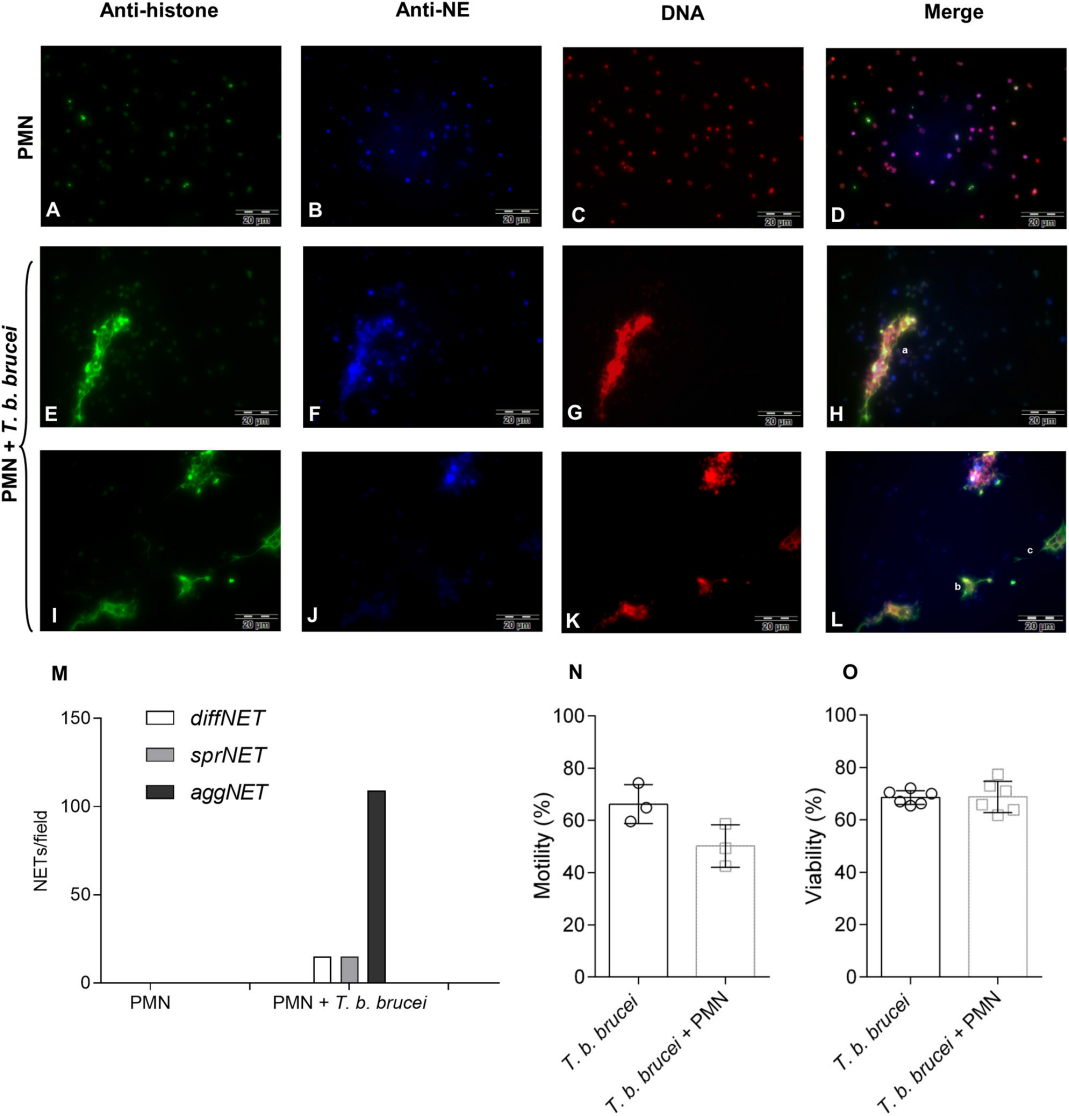
Immunofluorescence analyses of *T. b. brucei*-induced neutrophil extracellular traps (NETs) formation. Presence of DNA (**C,G,K**; red), histones (**A,E,I**; green), and neutrophil elastase (**B,F,J**; blue) in *T. b. brucei* induced NETs. **(D,H,L)** Depicts the merges of the three channels. **(M)** Demonstrates the cumulative frequency of the different phenotypes of observed NETs (i.e., *diff*NETs, *spr*NETs, and *agg*NETs) that were identified microscopically (*n* = 3). For more images showing the different NETs phenotypes please see Supplementary Figures 2, 3. Also, the percentage of motile and viable parasites was estimated **(N,O)** after 2 h of co-cultivation with PMN.

Different NETs phenotypes were detected in trypomastigote-exposed PMN. These NETs phenotypes have previously been described for other parasite species capable to induce NETs [(35, 36, 40); Figure 3 and Supplementary Figures 2, 3]. Correspondingly, *diff*NETs were identified as a complex of extracellular decondensed chromatin with a size of 15–20 μm diameter, *spr*NETs consisted of smooth and elongated web-like structures being composed exclusively of thin fibers with a diameter of 15-17 μm and *agg*NETs were characterized by a “ball of yarn” shape and sizes of more than 20 μm. Interestingly, interactions of *T. b. brucei* trypomastigotes with PMN mainly triggered *agg*NETs after 2 h of co-cultivation. Besides, also *diff*NETs and *spr*NETs were detected but at a minor proportion (Figure 3M).

### Bovine Neutrophil Extracellular Traps Slightly Decrease the Motility of *T. b. brucei* Trypomastigotes Without Affecting the Viability of the Parasite

To evaluate and quantify the effect of bovine PMN against the parasite, we evaluated the motility of *T. b. brucei* after 2 h of co-cultivation with PMN. Figure 3N depicts the motility of the parasite after co-cultivation with bovine PMN demonstrating that 50.17% of the parasites are motile against 66.24% of the trypomastigotes in the PMN-free condition. Also, in the same set-up, the viability of the trypomastigotes was evaluated. As is depicted in Figure 3O, activated bovine PMN are not able to kill *T. b. brucei* trypomastigotes, indicating that NETs can entrap in a non-lethal way a small number of parasites.

### 3D-Holotomographic Live-Cell Imaging of *T. b. brucei-*Mediated Neutrophil Extracellular Traps

**Figure 4:**
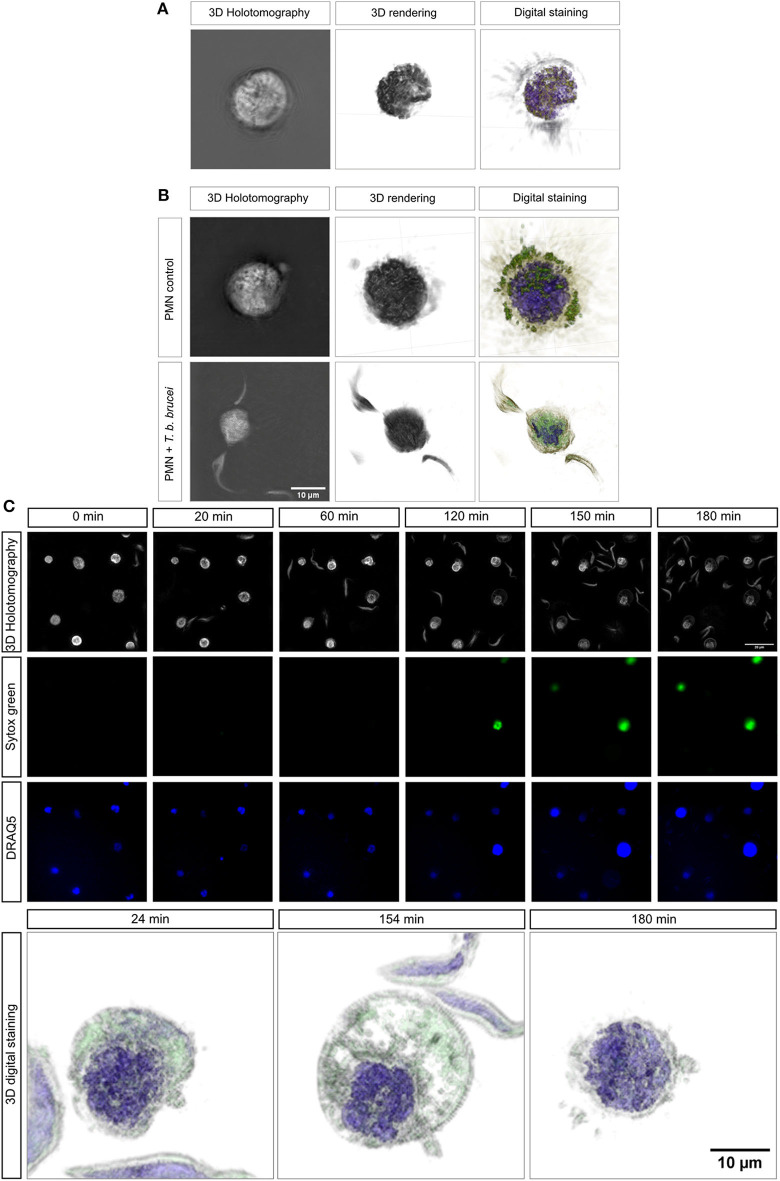
Live-cell 3D-holotomographic microscopy of PMN co-incubated with *T. b. brucei*. PMN were co-incubated with live parasites or vehicle in a conditioned imaging media. **(A,B)** Shows the 3D- holotomography image, 3D-rendering and -digital staining of bovine PMN alone or co-incubated with parasites, respectively. The digital staining is obtained based on refractive index (RI) values of the cell structures. In purple the segmented nuclei is depicted and in green vesicles and membrane-containing cell components are observed **(A,B)**. **(C)** Represents a time-lapse of stained PMN with Sytox green (green) and DRAQ5 (blue). 3D-digital staining shows representative morphological changes suffered by parasite-exposed PMN at 24, 154, and 180 min, respectively.

To be as close as possible to the *in vivo* situation, we performed 3D-holotomographic live-cell imaging (3D Cell Explorer®, Nanolive) to visualize the interactions between bovine PMN and *T. b. brucei* trypomastigotes. The morphology of a non-activated bovine PMN is illustrated in Figure 4A, complemented with 3D rendering and digital staining based on refractive index (RI) (Figures 4A,B), thereby allowing for the differentiation of some classical structures of these cells, such as a segmented nucleus (purple), granular cytosol, and plasmatic membrane (green). Additionally, Figure 4B illustrates an interaction of PMN and parasites.

To complement the results obtained by immunofluorescence microscopy in fixed cells, a live cell microscopy experiment was here performed, allowing a closer look to interactions and morphological changes. Figure 4C shows single images of the time-lapse experiment. The complete video of the live-cell imaging experiment can be found on the Supplementary Video 1. As depicted in Figure 4C, an increase on Sytox Green® signal (= extracellular DNA release; green) was observed after 120 min of co-culture. This signal was correlated with a change in nuclear shape as illustrated by DRAQ5 staining (blue). This finding confirms that *T. b. brucei* trypomastigotes indeed trigger DNA release. 3D-digital staining allowed for illustration of distinct morphological changes as protrusion and bubbling of bovine PMN membrane (Figure 4C; lower panel). Also, bovine PMN during confrontation with highly motile *T. b. brucei* shows nuclear expansion during the time and further lysis. Thus, especially cytoplasmic expansion and changes in nuclear shape were observed after 154 min whilst within the first 24 min of exposure, hardly any morphological change could be noted. The complete video registry of the 3D rendering of a unique cell is shown in Supplementary Video 2. Finally, after 180 min, we were able to detect the considerable expansion of PMN nuclei, thereby obviously reflecting the initiation of the NETotic process.

### Quantification of Neutrophil Extracellular Traps Formation via Nuclear Area Expansion-Based DNA Area and NETosis Analysis

**Figure 5:**
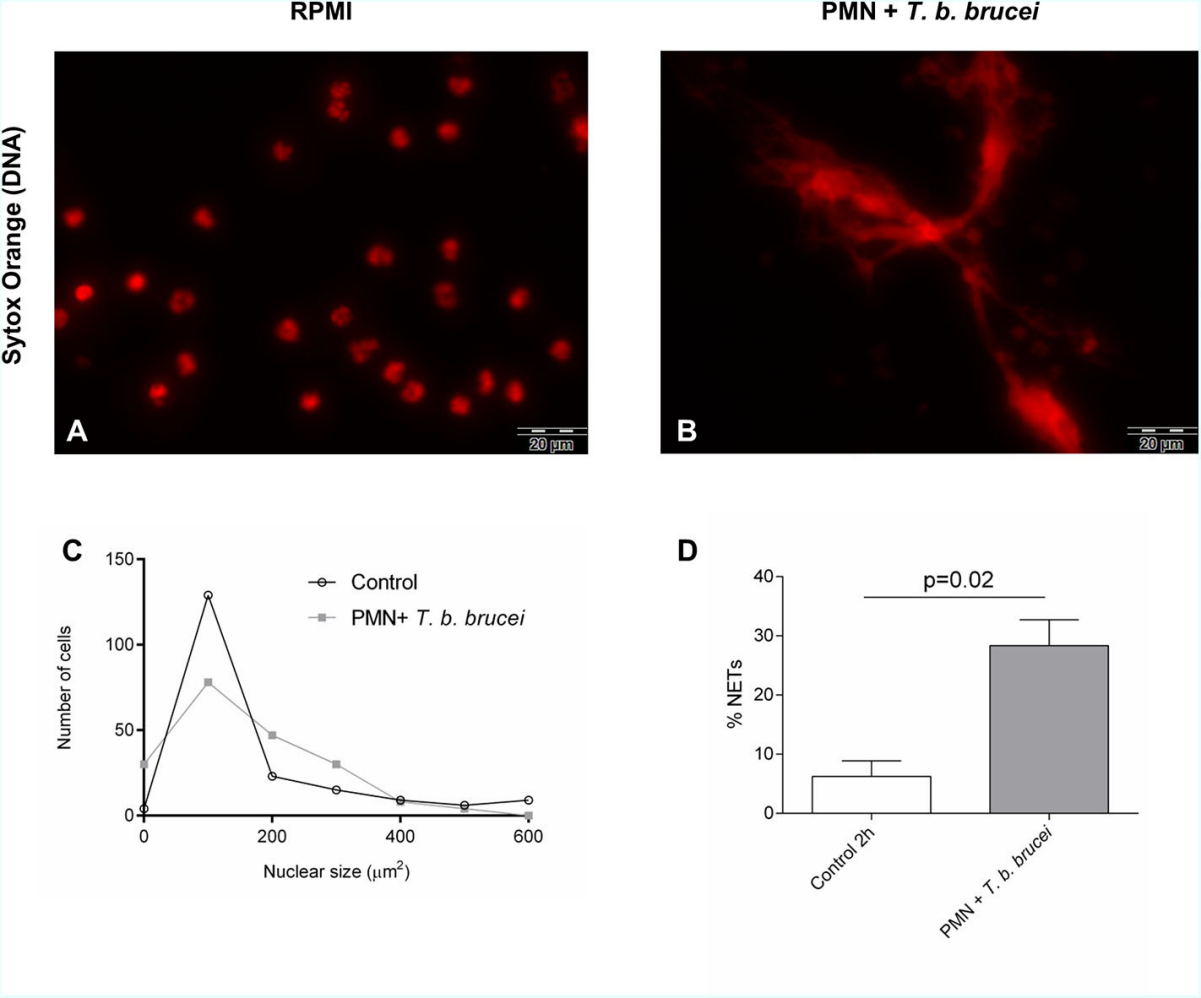
Interactions between bovine PMN + *T. b. brucei* triggered NETosis analyzed by nuclear expansion (NAE)-based quantification. Bovine PMN were incubated in cell medium alone or exposed to *T. b. brucei* on coverslips. **(A)** Corresponds to PMN alone (zoom) and **(B)** represents the co-cultivation of PMN + *T. b. brucei*. After fixation and staining of DNA with Sytox Orange (red), the NAE of 200–300 cells per condition were analyzed by ImageJ and DANA software **(C)**; the percentage of NETotic cells defined as PMN with values of nuclear area >90 μm^2^ was determined **(D)**. Statistical significance was defined by a *p < 0.05 in the Mann-Whitney test.

Nuclear area expansion (NAE) and chromatin decondensation is an early event of the NETs formation as described by (19). For this reason, we analyzed *T. b. brucei*-triggered NAE in PMN and estimated the percentage of cells undergoing NETs release using the software tool “DNA area and NETosis analysis” (DANA) as reported before (39) to guarantee an observer-independent estimation. To evaluate if exposure to *T. b. brucei* trypomastigotes indeed affected the DNA area of PMN nuclei after exposure, a total of 201 cells were analyzed for each experimental condition and the data were illustrated as frequency histograms (Figure 5C). However, we were unable to detect significant changes in mean values of the nuclear area within interactions between motile *T. b. brucei* and bovine PMN (Figure 5C) most probably resulting from high inter-donor variations, which are often reported for non-syngeneic beings. On the other hand, when the percentage of cells forming NETs was calculated by DANA, this approach documented induction of NETs formation by the parasites. Thus, 28.3% ± 3.57 of PMN released NETs upon trypomastigote exposure, compared to 6.25% ± 2.62 in non-exposed control cells (Figure 5D).

### *T. b. brucei-*Induced Neutrophil Extracellular Traps Formation Seems to Be Dependent on Purinergic Signaling

**Figure 6:**
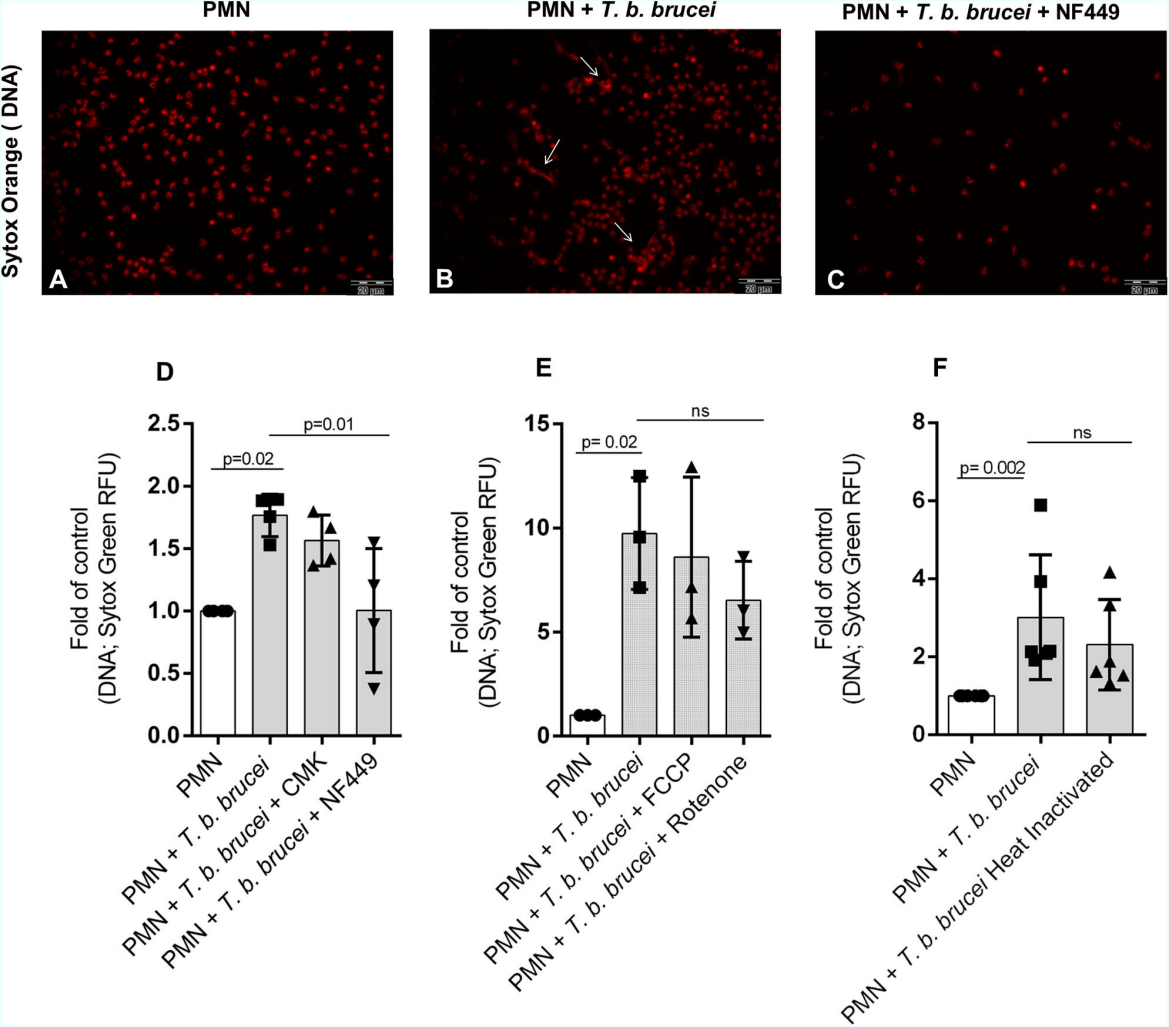
*T. b. brucei-*induced NETs in bovine PMN is depending of purinergic signaling. Bovine PMN (*n* = 4) were pre-treated for 30 min with NF449 (100 μM) and the NE inhibitor Suc-Ala-Ala-Pro-Val chloromethyl ketone (CMK; 1 mM) and then *T. b. brucei* trypomastigotes were added. **(A–C)** Shows microscopy images of cells stained with Sytox Orange (red). Besides, the release of DNA from bovine PMN was quantified using media that contains Sytox Green **(D)**. Additional experiments using mitochondrial inhibitors rotenone/antimycin A 5 μM and FCCP 0.5 μM were performed showing a partial non-significant inhibition of DNA release (**E**; *n* = 3). Finally, the DNA release induced by heat-inactivated (non-motile) trypomastigotes was evaluated, showing a decrease of nearly 26% on the quantity of released DNA, however, this tendency was non-statistically significant (**F**; *n* = 6). All values are represented as fold over the control. *p*-values indicated in the graphs (mean ± SD) were calculated using a non-parametric Kruskal-Wallis followed by Dunn's *post-hoc* test.

We also investigated the role of the purinergic signaling pathway to study whether *T. b. brucei*-triggered NETs release is an energy and ATP-dependent process. Therefore, bovine PMN were pre-treated with different inhibitors: 100 μM of NF449 as an inhibitor of purinergic receptors and CMK 1 mM as neutrophil elastase inhibitor. As an interesting finding, the pre-treatment of PMN with NF449 almost completely abolished parasite-triggered NETs formation when compared to non-treated controls (Figures 6A–D). However, blocking NE activity with CMK did not affect parasite-triggered NETs formation. These data indicate that *T. b. brucei*-induced NETs formation seems to be dependent on purinergic-mediated ATP binding but is seemingly independent of NE activity. Also, other inhibitors of key signaling pathways in NETs formation were tested. Interestingly, NADPHOX inhibitor (DPI); glycolysis inhibitor (2-deoxy-D-glucose) and PAD-4 inhibitor (Cl-amide) have no effect over NETs formation –measured as DNA extrusion- on bovine PMN confronted to *T. b. brucei* trypomastigotes (Supplementary Figure 4). It is important to mention that, as a limitation of our study the lack of positive controls in our inhibition experiments makes impossible to know if the inhibitors worked properly in our experimental settings.

### Evaluation of Mitochondrial Activity Inhibitors on *T. b. brucei*-Mediated NETosis

To evaluate if the mitochondrial activity is involved on the NETotic process, experiments were performed using the inhibitors rotenone mixed with rotenone/antimycin A (inhibitor of complex I and III, Agilent) and phenylhydrazone (FCCP, disruptor of mitochondrial membrane potential, Agilent). The treatment with rotenone/antimycin A resulted in partial, but no significant inhibition of the parasite triggered-NETs formation (Figure 6E).

### Heat Inactivation Does Not Hamper *T. b. brucei*-Triggered Neutrophil Extracellular Traps Release

To determine the influence on NETs formation of alive *T. b. brucei* trypomastigotes against immotile parasites we used heat-inactivated trypomastigotes. After 2 h of co-cultivation, we confirmed that alive trypomastigotes induced an increase in NETs release and the release of NETs by heat-inactivated parasites showed a non-significant decrease in comparison with live non-treated parasites (Figure 6F).

## Discussion

This study shows for the first time that bovine PMN cast NETs in response to motile *T. b. brucei* trypomastigotes *in vitro*. After the first description of NETs formation as pivotal innate immune effector mechanism against invasive bacteria in 2004 (7), several reports have confirmed the relevance of this ancient defense mechanism not only against bacteria but also against several protozoan and metazoan parasites (16, 30, 37). However, little is known on NETs triggered by the genus *Trypanosoma*. So far, only two studies report on *T. cruzi*-induced NETosis in human- (28), opossum- and canine-derived PMN (13). In the case of human PMN, it has been described that *T. cruzi*-mediated NETosis is triggered via TLRs, specifically by TLR 2 and TLR 4 (28).

In contrast to *T. cruzi, T. b. brucei* includes obligate extracellular stages (41) meaning that endogenous parasites are permanently exposed to leukocytes either present in blood/lymphatic stream or being attracted to *T. b. brucei-*infected tissues/organs *in vivo* (42, 43). Nonetheless, trypanosomes are well-known to effectively escape host cell immune responses. Consistently, *T. b. brucei* trypomastigotes are reported to constantly remodel their cell surface via viable surface antigens (VSA), explaining their ability to evade adverse immune reactions (42).

PMN display different effector mechanisms to combat parasites, such as phagocytosis, ROS production and NETosis (11, 16). Of note, ROS production seems essential for NETs extrusion (20, 21, 44). This process is linked to both, an increase in OCR and enhancement of proton efflux rates (PER) (45) which are necessary for proper NETs formation (22). Consistently, OCR and PER were found rapidly enhanced in bovine PMN after incubation with motile *T. b. brucei* trypomastigotes. These notorious increases were sustained for more than 240 min of stimulation, thereby suggesting considerable ROS production and induction of PMN-derived metabolic activities. We observed a fast increasing tendency in PMN confronted with *T. b. brucei* trypomastigotes in both, total ROS and extracellular superoxide. This tendency does not show statistical significance, mainly due to the low number of animals studied and inter-donor variation (38, 46). The OCR consumption was also increased in the presence of rotenone/antimycin A mixture, as an indirect way to measure the contribution of NADPHOX and mitochondrial complexes in the observed increase. Our results show that the mitochondrial activity, critical for other PMN functions as chemotaxis, phagocytosis, and respiratory burst (47) has a partial involvement also in extracellular DNA release from bovine PMN.

To measure morphological changes during the early phase of the NETotic process, we performed DANA, as described before (39). DANA-based analysis of NAE in exposed PMN is accepted as an indicator of early NETs formation process as demonstrated previously (19, 48, 49). In this study, we used DANA to better characterize interactions of PMN and *T. b. brucei* trypomastigotes. This approach allowed NETs-forming PMN quantification. However, in contrast to other reports, we did not find significant differences among the nuclear area after undergoing cytoplasmic changes at 2 h of parasite confrontation. This discrepancy may be because PMN in our study have been stimulated with vital parasites and not by classical NETs inducer molecules, such as phorbol myristate acetate (PMA), zymosan, ionophores, cytokines/chemokines, or lipopolysaccharide (LPS) (15, 50, 51). By using the default threshold of DANA for the nuclear area of 90 μm^2^, we were able to detect a significant increase in the percentage of NETs-forming cells triggered by *T. b. brucei* trypomastigotes (Figure 5) corresponding well to results obtained by direct visualization. Also, it must be noted that DANA was used successfully in the human and murine system, with the present report being the first attempt to quantify NETs formation by DANA in bovine PMN.

To confirm our observations in living cells, we additionally performed live-cell imaging using a 3D Cell Explorer® microscope (Nanolive) and an Ibidi® top-stage chamber (Ibidi) to keep the temperature and CO_2_ atmosphere conditions stable. Overall this technical approach allowed for the generation of 3D rendering and digital staining-based images of PMN in different activation stages and activities, i.e., resting PMN, activated ones, PMN in close contact to trypomastigotes-, and/or cells casting NETs. Classical morphological features of bovine PMN were obtained, such as the abundant presence of cytoplasmic granules, polymorphic nuclei, attachment activity, and laminopod formation (52–54). Overall, co-culture of bovine PMN and trypomastigotes induced an increase of nuclear size after 120 min post-incubation strongly suggesting NETotic process. Moreover, we were able to detect nuclear degeneration after 180 min post-parasite exposure by 3D-digital staining, which may indicate a late NETotic process, as previously reported (19).

Interestingly, *T. b. brucei*-induced NETs formation resulted in different phenotypes of NETs, which is in accordance to other reports on parasite-induced NETosis (12, 35, 36). Thus, *T. b. brucei-*stimulated PMN predominantly cast *agg*NETs and *spr*NETs; *diff*NETs were also extruded but to a minor extent, corresponding well to previous findings on large-sized *D. immitis* larvae-induced NETs (36). Immunopathological implications of this observations are related to the fact that *agg*NETs are proposed to have anti-inflammatory effects via sequestration and detoxification of histones (55) and the proteolysis of cytokines and chemokines (56) whereas *spr*NETs and *diff*NETs are pro-inflammatory in the early phase of the immune response (57). In this context, loss of the continuity of the blood-brain barrier is necessary for the increased leukocyte counts in cerebrospinal fluid (CSF) and the presence of *T. b. brucei* in the CSF of affected animals (58). Considering this, NETs induce endothelial damage by the presence of histones (43, 59, 60) and provides a scaffold for the alternative complement pathway (61). Moreover, it was reported that *agg*NETs involve a higher number of PMN, suggesting a rather marked PMN attraction and activation after the first contact with these rather large and motile haemoflagellates. Interestingly, PMN can distinguish the sizes of pathogens and selectively release NETs in response to them (62). Taking PMN sensing capacities into account, we here hypothesize that not only size but also movements might influence in *T. b. brucei*-triggered NETs, based in observations of tiny and immotile bacteria or viruses. Interestingly, previous reports showed that movement in flagellated bacteria (63) and also on parasites (36) is important for NETs release. Even so, viability and motility experiments were performed, showing that in our experimental conditions triggered NETs did not promote the killing of this parasitical stage, helping us to hypothesize that NETs have a role on immobilization than the viability of the parasite (12, 36).

Purinergic receptors are involved in several activities of PMN, such as chemotaxis, phagocytosis, oxidative burst, apoptosis, and degranulation (31, 64). In line, it was recently reported that extracellular ATP regulates PMN chemotaxis via P2Y_2_ receptors and that P2Y receptors are involved in PMN adhesion to the endothelium (31). Even so, studies have demonstrated that *T. cruzi* can induce calcium intake using the Pannexin-1 channel, which allows cells to release ATP to the environment. This could help to trigger the PMN activation, considering the chemotactic effect (65). In this study, we found that purinergic receptors indeed play a role in *T. b. brucei*-induced NETs formation, since the inhibition of this receptors by using NF449 decreased NETs formation to a considerable extent, which is in accordance to previous parasite-based reports (23, 66). However, since we used a concentration 10–100 times higher that some of these previous reports, we cannot discard an influence of G protein-coupled receptors (GPCRs). Further experiments will be performed to establish which purinergic receptors are involved in the activation of bovine PMN. On the other hand, treatments with rotenone/antimycin A and FCCP, both inhibitors of mitochondrial function, partially reduced the NETs formation, suggesting a partial role of mitochondrial activity in *T. b. brucei* induced NETs release. Interestingly, a critical role for the mitochondrial function of bovine PMN in response to platelet-activating factor (PAF) was recently demonstrated (64). In addition, no inhibition was observed when the other inhibitors as CMK (elastase inhibitor), DPI (NADPHOX inhibitor), Cl-amide (PAD4 inhibitor) were used. Since we did not demonstrate the activity of our inhibitors by the use of positive controls, we cannot do conclusive statements on this regard. Moreover, the possibility of that PAD4 and elastase have redundant functions on NETs formation induced by *T. b. brucei* cannot be discarded.

NETs are mainly composed of decondensed chromatin alongside with citrullinated nuclear histones and enzymatic granular components, such as NE, MPO, lactoferrin, calprotectin, LL37, pentraxin, proteinase 3 (P3) and cathepsin G (CG) among others (19, 67). Current co-localization experiments on PMN-derived DNA being decorated with histones and NE on *T. b. brucei-*triggered extracellular structures proved that NETs are induced by trypomastigotes. PMN activation and extrusion of nuclear DNA was also documented by live cell 3D-holotomographic microscopy indicating that NETs indeed are formed as a response to large and motile pathogens when compared to virus, bacteria or fungi. Furthermore, our data showed that almost a third of PMN (28.3% ± 3.57) being confronted with *T. b. brucei* stages released NETs.

Overall, we here present novel data on PMN-derived NETs formation against highly motile and rather large extracellular *T. b. brucei* trypomastigotes (compared to bacteria, fungi or virus) as part of the host innate immune responses *in vitro*. Considering blood/lymph localization of *T. b. brucei in vivo*, parasite entanglement via NETs release could be of particular importance since immobilized trypomastigotes might become potential targets for other leukocytes being attracted to sites of parasite entrapment or intravascular NETs release. The pivotal role of purinergic-dependent signaling is also postulated but needs further investigation since other members of this receptor family might as well-participate in *T. b. brucei*-mediated NETs release. However, the complete role of NETs-derived effects on these euglenozoan parasites as well as possible NETs-derived damage on exposed endothelium (60) of lymph/blood vessels *in vivo* is not yet clear and will be addressed in the near future. The same holds for the possible role of *T. b. brucei*-triggered NETs release in the immunopathology of AAT such as intravascular coagulopathies or vascular permeabilization.

## Data Availability Statement

The raw data supporting the conclusions of this article will be made available by the authors, without undue reservation.

## Ethics Statement

The animal study was reviewed and approved by Regierungspräsidium Giessen; 147 A9/2012; JLU-No.521_AZ (Ethic Commission for 146 Experimental Animal Studies of Federal State of Hesse).

## Author Contributions

AT, CH, and IC conceptualization and supervision. DG investigation (PMN isolation, *T. b. brucei* cell culture, immunofluorescence, DANA analyses, and inhibition experiments), formal analyses, data visualization, and wrote the original draft. IC carried out the investigation (SEM, Nanolive, Seahorse) and data visualization. ZV obtained confocal microscopy images, performed Nanolive video analysis, and data visualization. CP provided the *T. b. brucei* cell culture for this study. RB and PA conceptualization of inhibition experiments. IC, PA, RB, AT, and CH reviewed the manuscript. CH and AT funding acquisition. All authors contributed to the article and approved the submitted version.

## Conflict of Interest

The authors declare that the research was conducted in the absence of any commercial or financial relationships that could be construed as a potential conflict of interest.
