# Supplementary material for: Trypanosoma brucei brucei Induces Polymorphonuclear Neutrophil Activation and Neutrophil Extracellular Traps Release
Source: Front Immunol. 2020 Oct 22;11:559561. doi: 10.3389/fimmu.2020.559561 (PMC7649812; doi:10.3389/fimmu.2020.559561)
Supplement: Supplementary file 3 [file Image_1.PDF]

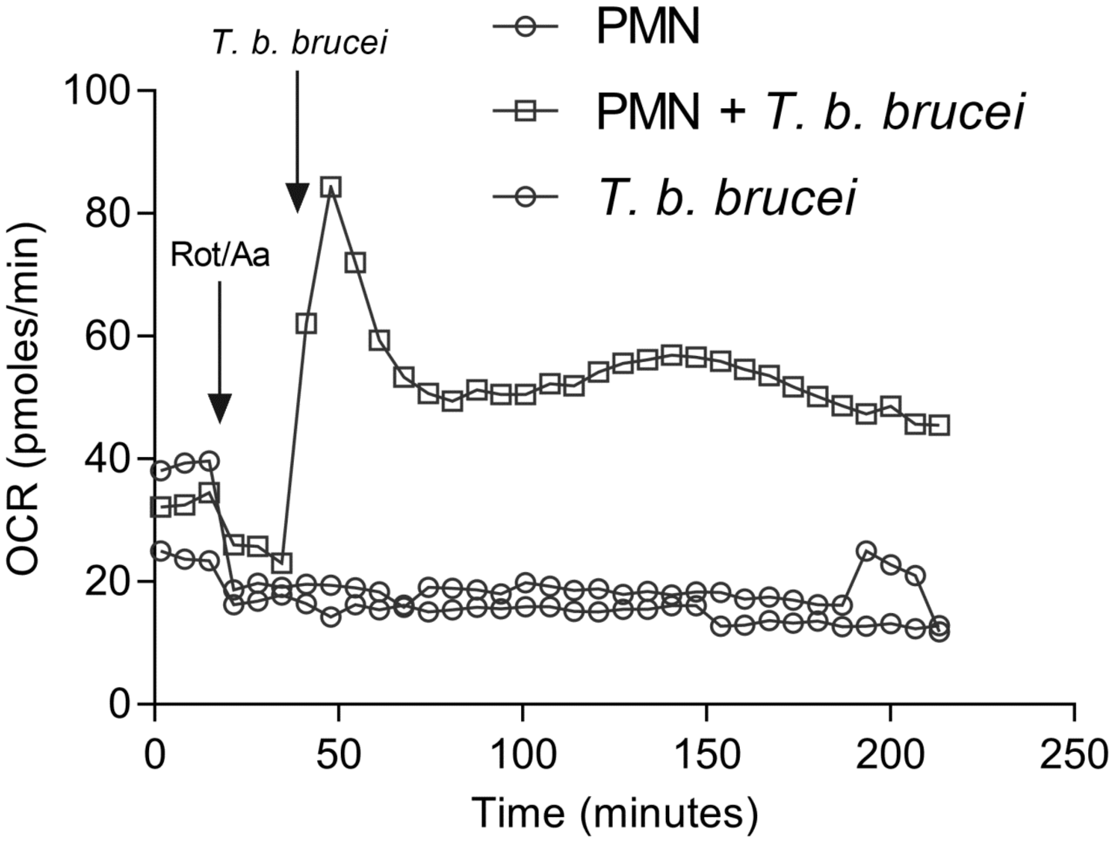

Fig S1. *T.b. brucei* does not increase the OCR in the experimental conditions used in this report. Typical registry obtained from an extracellular flux analyser (Seahorse) showing the oxygen consumption rate (OCR) of PMN alone, PMN + *T. b. brucei* and *T. b. brucei* alone. Injection of 6 x 10^5 parasites does not increase the OCR.

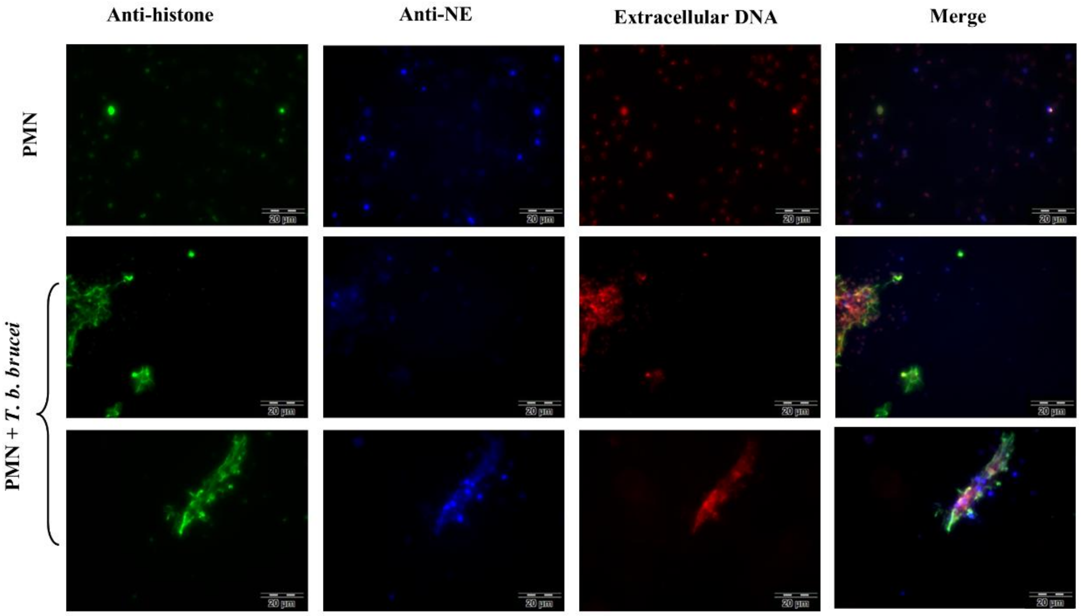

Fig S2. Immunofluorescence analyses on *T. b. brucei* induced neutrophil extracellular trap (NET) formation

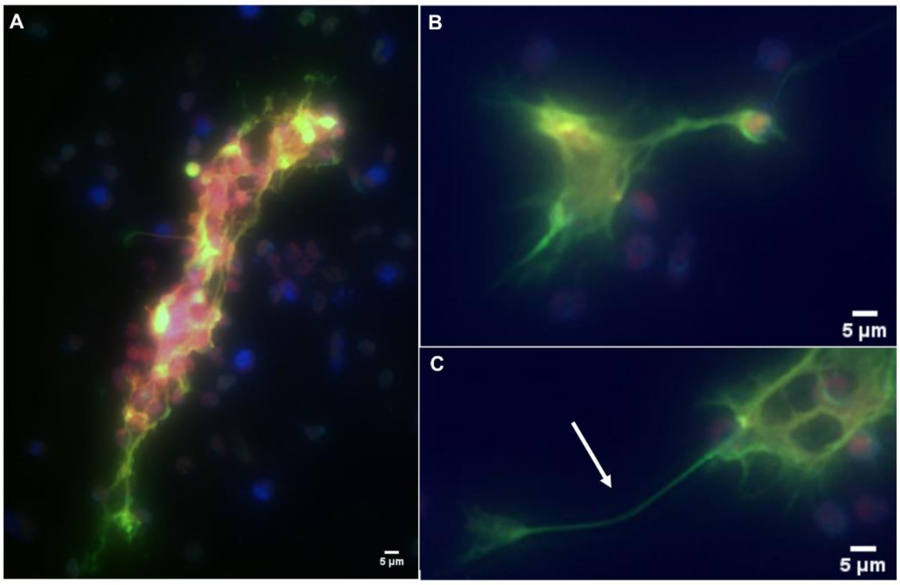

Fig S3. *T. b. brucei* trypomastigotes trigger different phenotypes of NETs. **(A)** *agg*NETs*,* characterized by having a "ball of yarn" shape and sizes of more than 20 µm. **(B)** *diff*NETs, formed as a complex of extracellular decondensed chromatin with a size of 15-20 µm diameter. **(C)** *spr*NETs (arrow), described as a smooth and elongated web-like structures being composed exclusively of thin fibers with a diameter of 15-17 µm.

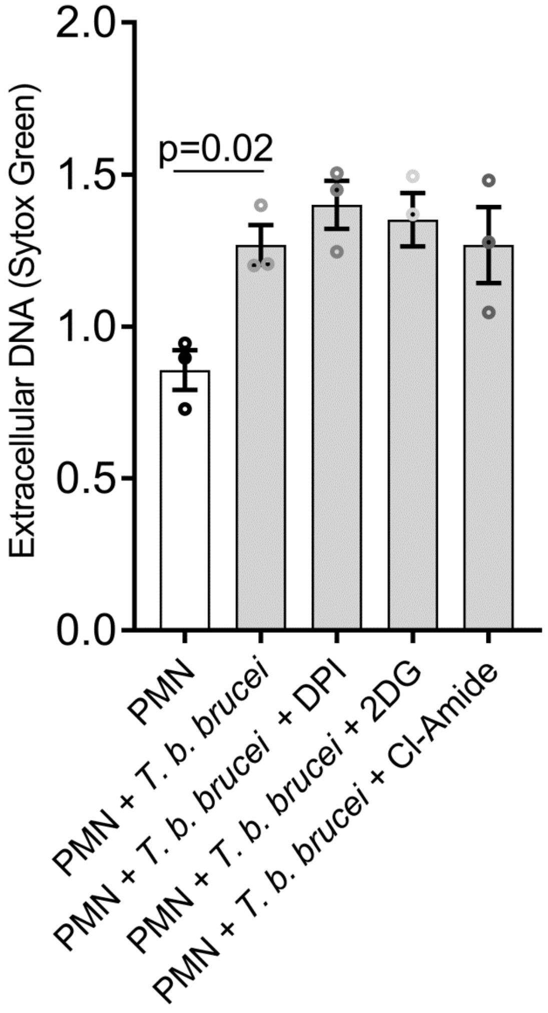

Fig S4. Effect of different inhibitors on extracellular DNA release from bovine PMN induced by *T. b. brucei*. 2 x 10^5 bovine PMN were incubated from 30 min at 37°C with DPI 10 µM, 2DG (2-deoxyglucose) 50 mM and Cl-amide 100 µM. Then, 6 x 10^5 *T. b. brucei* were added and incubated for 2 hours in the presence of 5 µM Sytox green. Fluorescence intensity was measured in a Varioskan microplate reader at an excitation wavelength of 504 nm and an emission wavelength of 523 nm. Values represent mean ± SD of normalized fluorescence values. p value corresponds to a ANOVA analyses followed by a Dunn´s post-hoc test considering the PMN+*T. b. brucei* condition as control.
